# Identification of baboon microRNAs expressed in liver and lymphocytes

**DOI:** 10.1186/1423-0127-17-54

**Published:** 2010-07-01

**Authors:** Genesio M Karere, Jeremy P Glenn, John L VandeBerg, Laura A Cox

**Affiliations:** 1 Department of Genetics, Southwest Foundation for Biomedical Research, San Antonio, 7620 NW Loop 410, TX 78227, USA; 2 Southwest National Primate Research Center, Southwest Foundation for Biomedical Research, San Antonio, 7620 NW Loop 410, TX 78227, USA

## Abstract

**Background:**

MicroRNAs (miRNAs) are small noncoding RNAs (~22 nucleotides) that regulate gene expression by cleaving mRNAs or inhibiting translation. The baboon is a well-characterized cardiovascular disease model; however, no baboon miRNAs have been identified. Evidence indicates that the baboon and human genomes are highly conserved; based on this conservation, we hypothesized that comparative genomic methods could be used to identify baboon miRNAs.

**Methods:**

We employed an *in silico *comparative genomics approach and human miRNA arrays to identify baboon expressed miRNAs in liver (n = 6) and lymphocytes (n = 6). Expression profiles for selected miRNAs in multiple tissues were validated by RT-PCR.

**Results:**

We identified *in silico *555 putative baboon pre-miRNAs, of which 41% exhibited 100% identity and an additional 58% shared more than 90% sequence identity with human pre-miRNAs. Some of these miRNAs are primate-specific and are clustered in the baboon genome like human miRNA clusters. We detected expression of 494 miRNAs on the microarray and validated expression of selected miRNAs in baboon liver and lymphocytes by RT-PCR. We also observed miRNA expression in additional tissues relevant to dyslipidemia and atherosclerosis. Approximately half of the miRNAs expressed on the array were not predicted *in silico *suggesting that we have identified novel baboon miRNAs, which could not be predicted using the current draft of the baboon genome.

**Conclusion:**

We identified a subset of baboon miRNAs using a comparative genomic approach, identified additional baboon miRNAs using a human array and showed tissue-specific expression of baboon miRNAs. Our discovery of baboon miRNAs in liver and lymphocytes will provide resources for studies on the roles of miRNAs in dyslipidemia and atherosclerosis, and for translational studies.

## Background

MicroRNAs (miRNAs) are endogenous, small (~22 nucleotides), non-coding RNAs that are transcribed by RNA polymerase II from intergenic, intronic or exonic regions of the genome [1]. Primary miRNA (pri-miRNA) transcripts are processed into precursor miRNAs (pre-miRNAs) in the cell nucleus by Drosa and Pasha protein complexes [2-4]. The pre-miRNAs are exported by exportin-5 to the cytoplasm [5,6] where an RNase III endonuclease, Dicer, cleaves the hair-structure in the pre-miRNA into mature doubled-stranded miRNAs [5]. The single stranded 5' terminus of the mature miRNA, is recruited into the RNA-induced silencing complex (RISC) ([7]. Guided by RISC, the miRNAs silence gene expression by degrading target mRNA when there is complete base-pairing, or by inhibiting translation when there is imperfect binding to the 3' untranslated region (UTR) [8,9]. Some miRNAs also bind to 5' UTRs [10]. miRNAs exhibit temporal and spatial expression patterns and are implicated in diverse cell functions for development, proliferation and differentiation [2,11,12]. Moreover, miRNAs show aberrant expression in diseases such as cancer, diabetes and cardiac diseases [13-17]. Recent studies indicate that the up-regulation of miR-335 and 122 is associated with lipid metabolism in obese mice [18,19]. This suggests that miRNA dysregulation may play a role in disease phenotypes and that miRNAs may be important biomarkers for disease diagnosis [20,21].

miRNAs are highly conserved across species, particularly in the first 8 nucleotides (nts) at the 5' end known as the 'seed region' [22,23]. Using the conserved regions of miRNAs, computational analyses have augmented the prediction of miRNAs in many different species. As of September 2009, 10,883 miRNAs have been deposited in the microRNA database miRBase (Release 14: http://www.mirbase.org) [24], including 750 human, 604 Chimpanzee and 483 rhesus macaque miRNAs. In addition multiple miRNAs are conserved as clusters in genomes, some of these clusters have common functional roles, such as the testicular oncogenic miR371/373 human cluster [25]. The evolutionary conservation of miRNAs among species suggests that miRNAs have conserved biological functions.

The baboon is a well-characterized model for human biomedical studies including cardiovascular disease, however no baboon miRNAs have been identified and reported in the miRBase. In the present study we compared human precursor miRNA (pre-miRNA) sequences with draft baboon genome sequence data to identify putative baboon miRNAs. After identifying the baboon miRNAs *in-silico*, we determined expression profiles of baboon liver and lymphocytes miRNAs using a human miRNA microarray. miRNA expression profiles for select miRNAs in baboon tissues were validated using RT-PCR. Our results indicate that cross-species sequence alignment can be used to identify putative miRNAs in an unannotated genome. In addition, these results show the miRNAs that are expressed in baboon liver and lymphocytes and the differences in these miRNA expression profiles. The findings from these studies are relevant to future studies of the roles of miRNAs in dyslipidemia and atherosclerosis; liver is a primary target organ for cardiovascular disease, whereas lymphocytes are an easily accessible diagnostic sample in humans.

## Methods

### *In silico *identification of putative baboon miRNAs

Human pre-miRNA sequences were accessed from the University of California, Santa Cruz (UCSC) Genome Browser [26,27] utilizing the Table function for SNO/miRNAs [28]. The human pre-miRNA data in the Genome Browser are from the miRBase Sequence Database at the Wellcome Trust Sanger Institute [29,30]. Human pre-miRNA sequences were used to query the NCBI trace archives of the *Papio hamadryas *whole genome sequence using the BLASTN program http://blast.ncbi.nlm.nih.gov/Blast.cgi. BLAST alignment was optimized for highly similar sequences. Algorithm parameters included automatically adjusting for short input sequences, an expected threshold of 10, word size of 28, match/mismatch scores of 1-2, linear gap costs, and regional low complexity filtering. In addition, baboon to human sequence alignments were filtered based on baboon sequence quality scores greater than 50. Predicted baboon pre-miRNAs are shown in Additional File 1.

### Identification of baboon pre-miRNA genomic clusters

Human genomic DNA regions containing pre-miRNA clusters were identified by UCSC genome browser. Genomic DNA for each cluster was aligned with the draft assembly of the baboon genome sequence in the trace archive http://blast.ncbi.nlm.nih.gov/Blast.cgi using the BLAST alignment tool [31]. To validate baboon draft assembly alignment, the baboon genomic DNA region was aligned against the human genome using the BLAT alignment tool [27]. In addition, human genomic DNA was aligned with the rhesus genome using the BLAT alignment tool.

Baboon genomic regions were aligned to regions of the other species by searching for homologous human miRNAs in the Baboon Test Genome Browser Gateway hosted by the UCSC http://genome-test.cse.ucsc.edu. BLAST was then used to search for baboon DNA sequences in the human genome for homologous region. Regional tracks of chimpanzee, rhesus, mouse and rat are also presented.

### Tissue Collection

All procedures were approved by the Southwest Foundation for Biomedical Research (SFBR) Institutional Animal Care and Use Committee and conducted in Association for Assessment and Accreditation of Laboratory Animal Care approved facilities. Liver biopsies and blood were collected from six baboons. Baboons were sedated with ketamine (10 mg/kg), given atropine (0.025 mg/kg) and intubated. Anesthesia was induced and maintained with isoflurane (1-2%). Blood pressure was measured by automated arm cuff (Collin) and oxygen saturation, heart rate, and respiration was monitored by pulse oximetry. A Southwest National Primate Research Center staff veterinarian collected biopsies. During post biopsy recovery analgesia was provided in the form of Stadol, 0.15 mg/kg, bid, for 3 days and ampicillin, 25 mg/day for 10 days. Liver, testis, femoral and coronary arteries, omental fat, and cerebrum were also collected opportunistically from one baboon after euthanization at necropsy. Tissue samples were quick frozen in liquid N_2 _and stored at -80°C. Lymphocytes were isolated from blood and stored at -80°C.

### Sample preparation

Total RNA was isolated from liver (n = 6) and lymphocytes (n = 6) of adult baboons and also isolated from testis, femoral and coronary arteries, omental fat, and cerebrum of an adult baboon using RNeasy kit (Qiagen) according to the manufacturer's protocol. Fresh baboon tissues were snap-frozen in liquid nitrogen and stored at -80°C until RNA was extracted. RNA was quantified using the protocol in the RiboGreen kit (Invitrogen). A standard curve was created from known concentrations of serial diluted rRNA and used to interpolate and determine the concentrations of RNA from the baboon samples.

### miRNA expression profiling

Baboon miRNAs were hybridized to a miRNA Beadchip Human Illumina Beadchip array version 2) containing 1,146 probes following the manufacturer's protocol http://www.illumina.com/technology/microrna_assay.ilmn. Briefly, 500 ng of total RNA was polyadenylated using a biotinylated oligo-dT primer, containing a universal PCR primer site at 5'end. Biotinylated cDNAs were generated by reverse transcription and hybridized to miRNA-specific oligos. Each oligo contains a 5'-end universal PCR priming site, an address sequence complementary to the capture sequence on the array bead and a 3' end microRNA-specific sequence. After hybridization, the mixture was bound to streptavidin-containing paramagnetic particles and unhybridized mixture washed off. Using a pair of universal primers, the hybridized mixture was amplified and a single-strand complementary to the array sequence fluorescently labeled. The labeled PCR products were hybridized to the array capture sequence attached to the array beads. Using a BeadScan reader (Illumina), array signal intensities were measured in duplicate using the embedded channels. The signal intensity corresponds to the quantity of respective miRNA in a sample.

### Data analysis

Data analysis was performed using a BeadStudio software (Illumina version 3.1.3.0). The miRNA intensity data were filtered by applying a detection threshold of p < 0.05, which corresponds to the mean signal intensity from each probe that is significantly different from the mean of a baseline control probe. The analyzed data was up-loaded into a spreadsheet and further analysis performed. The mean detection values from a set of 12 redundant oligos probing single miRNA were averaged and signal intensities with detection p-values < 0.05 were considered expressed.

### Design of primers for miRNA RT-PCR

Primer pairs and miRNA sequences used for RT-PCR are presented in Additional file 1. For the primer design, we followed previous description [32]. Primers for the RT-PCR included a stem-loop RT primer containing 4-6 nts at the 3' end complementary to the miRNA molecule, a miRNA-specific forward primer, and a universal reverse primer. Synthetic miRNA oligonucleotides were purchased from Integrated DNA Technologies (IDT).

### Reverse Transcription reactions

For the RT-PCR, 80 ng of RNA was reverse transcribed to generate miRNA specific first-strand cDNA. A 20 ul RT reaction also included 1× PCR buffer, 0.5 mM dNTP, 0.5 U RNase inhibitor, 1.5 mM MgCl_2_, 1 uM reverse transcription primer, 0.5 uM DTT and 0.25 U of Superscript III reverse transcriptase. The RT-PCR mixture was incubated in an AB 9700 Thermocycler for 30 min at 16°C, 30 min at 42°C, 5 min at 85°C and held at 4°C. Controls included a master mix with no reverse transcriptase.

### PCR

Two micro liters of miRNA specific cDNA was amplified in AB 9700 Thermocycler in a 96-well plate using the following profile: denaturation for 5 min at 95°C, followed by 35 cycles of 30 sec at 94°C, 45 sec at 60°C, 30 sec at 72°C and final extension for 7 min at 72°C. Each PCR reaction (20 ul) contained 1× PCR buffer, 0.8 mM dNTP, 1 uM of paired primers, 3.5 mM MgCl_2 _and 0.25 U ExTaq polymerase (Takara Bio Inc.). PCR products in a denaturing loading dye were incubated at 95°C for 5 min, chilled on ice and size-fractionated on a 3% agarose gel in 1× TBE buffer at 6 V/cm. The gel was stained with ethidium bromide before visualization under UV light. For the PCR, the negative control consisted of a master mix with no cDNA.

## Results

### Prediction of Baboon genome miRNAs

**Figure 1:**
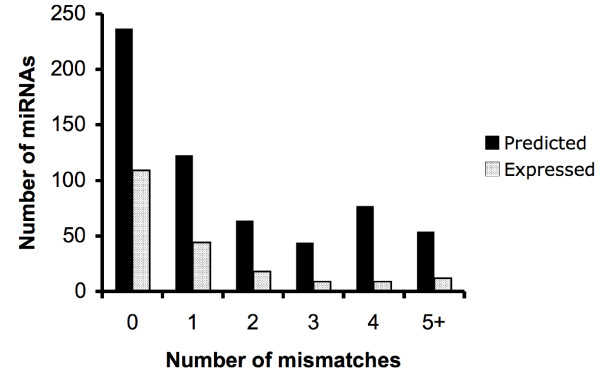
**The number of mismatches compared to the number of predicted and expressed baboon miRNAs**. The x-axis indicates the number of nucleotide differences when comparing baboon and human pre-miRNA sequences; the y-axis denotes the number of predicted (black) and expressed (gray) pre-miRNAs.

Alignment of published human precursor miRNA sequences from the miRBase database http://www.mirbase.org with the baboon genome sequences predicted 555 baboon miRNAs. Information on the predicted baboon miRNAs including names, genomic coordinates, length, mismatches and percent identity with human pre-miRNA sequences are available in Additional file 2. The length of the predicted pre-miRNAs ranged from 28 to 150 nts with an average of 81 nts. Only 54 (9.7%) of the total predicted miRNAs had more than 4 bp mismatches between human and baboon sequences (Figure 1). Of the 555 predicted baboon miRNAs, 227 (40.9%) shared 100% sequence identity with the human pre-miRNAs and 319 (57.5%) had greater than 90% and less than 100% identity with human pre-miRNAs (Table 1).

**Table 1:** Conservation of pre-miRNAs sequences between human and baboon.

Alignment identity (%)	100	99-98	97-96	95-94	93-92	91-90	89-88	87	Total
Number miRNAs	227	124	88	61	33	13	8	1	555

Percent miRNAs	40.9	22.3	15.9	11	5.9	2.3	1.4	0.2	100

### miRNA expression profiling

Expression profiling of baboon liver (n = 6) and lymphocyte (n = 6) miRNAs using a miRNA microarray detected expression of 494 miRNAs (Table 2 and Additional file 3). Sixty-eight (13.8%) were expressed only in lymphocytes and 8 (1.6%) were expressed only in liver. Of the 494 expressed miRNAs, 205 (41.5%) were predicted by *in-silico *analysis (Table 3), while 289 are likely new baboon miRNA identified through the human miRNA array.

**Table 2:** Summary of miRNA expression profiling for baboon liver and lymphocyte RNA.

miRNA Expression	Number	Percent
Both Liver and Lymphocytes	418	84.6
Liver Only	8	1.6
Lymphocytes Only	68	13.8
Total miRNAs Expressed	494	100.0

**Table 3:** Summary of miRNAs predicted and expressed in baboon liver and lymphocyte RNA.

miRNA Expression	Number	Percent
Both Liver and Lymphocytes	189	92.0
Liver Only	2	1.0
Lymphocytes Only	14	7.0
Total miRNAs Expressed	205	100.0

### Validation of expressed miRNAs

**Figure 2:**
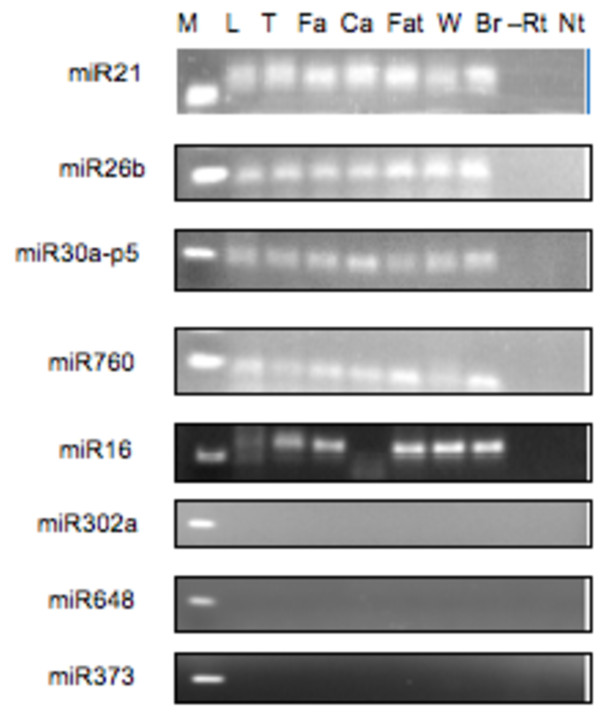
**miRNA expression in baboon tissues**. miRNA RT-PCR products generated by stem-loop RT-PCR were size-fractionated in a 3% agarose gel. Samples include: M: 50 bp marker, L: liver, T: testis, Fa: femoral artery, Ca: coronary artery, Fat: omental fat, W: lymphocytes, Br: brain, -Rt: RT control without reverse transcriptase, Nt: non-template control.

Validation of liver and lymphocyte miRNA microarray expression profiles by Reverse Transcription-PCR (RT-PCR) in liver and lymphocytes was performed for a subset of expressed and undetected miRNAs. We confirmed expression of miR21, 26b, 30a-5p, 760, and 16-1 (Figure 2) and lack of detectable expression for miR302a, 648, and 373. Expression profiles for additional tissues (testis, femoral and coronary arteries, omental fat and cerebrum) showed expression of miR21, 26b, 30a-5p and 760, and tissue specific expression of miR16-1. miRNAs that did not show a detectable signal on the miRNA array did not show a product using RT-PCR validating the specificity of the miRNA array data for both the expressed and undetected miRNAs.

### miRNA gene clusters

**Figure 3:**
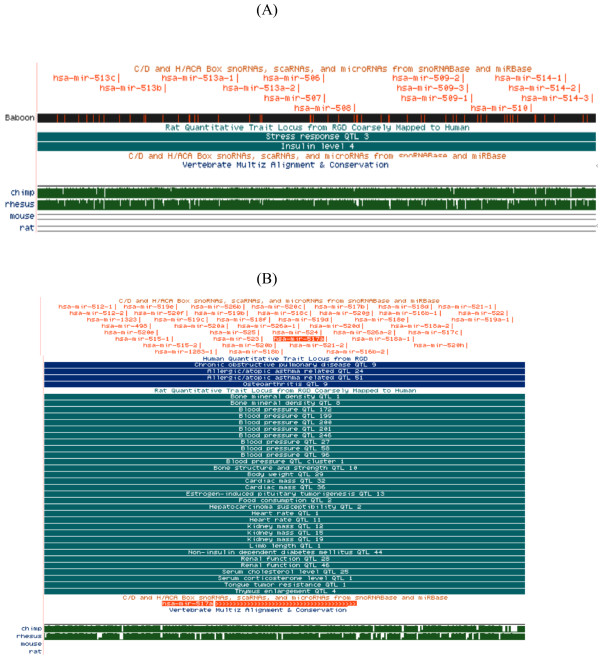
**Conservation of human pre-miRNA cluster on human a) chr X, and b) chr 19 with baboon, chimp, rhesus, mouse and rat**. miRNA clusters are shown using the UCSC graphical display. The pre-miRNAs are shown in the C/D and H/ACA track; conservation between baboon and human DNA is shown in the track labeled "Your Sequence from Blat Search"; Quantitative Trait Loci mapping to the chromosomal region is shown in the "Quantitative Trait Locus" track; and conservation between baboon and human, chimp, rhesus, mouse and rat are shown and a summary of overall conservation for the genomic regions (2a. chr X: 146,059,514-146,180,617 and 2b. chr 19: 58,831,904-58,961,623) are shown in the "Conservation" tracks.

Previous studies have demonstrated that miRNA genes may exhibit clustering in the genome [33] and that some clusters are primate specific [34]. Our analyses confirm that the miRNA clusters on chromosome (chr) 19 and X are conserved in primates including rhesus macaque and baboon but not in non-primate mammals such as rat and mouse (Figure 3). Both clusters are localized at a subtelomeric region on the q-arm that displays evolutionary conservation among 17 vertebrate species. The miRNA cluster on chr 19 is located at 58,831,904-58,961,623 bp, a region harboring human and rat QTLs including a QTL encoding serum cholesterol trait. The chromosome X cluster is localized to 146,059,514-146,180,617 bp and includes QTLs encoding insulin and stress responses. Although clustered miRNA family members tend to have similar expression patterns, in this study miR302a* was expressed in baboon liver and lymphocytes while 302a was not detected. We also observed that some chr. 9 cluster members (miR521, 520e, 373*, 373, 367) were not detected in both baboon liver and lymphocytes; however, miR514 cluster member on chr X was expressed in the liver and not detected in lymphocytes.

## Discussion

Previous studies have identified and quantified miRNAs in various species and in some cases shown that miRNA influences disease susceptibility. For quantification and identification of miRNAs, cloning and sequencing, northern blotting and primer extension methodologies have been employed. Recently, arrays have been used for high throughput quantification of miRNA expression in different normal and diseased tissues, e.g. neuronal differentiation [35,36]. The principle objective of this study was to identify and quantify baboon miRNAs expressed in liver that may be relevant to lipid metabolism in baboon and determine if these liver miRNAs could also be detected using an easily accessible RNA source, lymphocytes. Due to the high degree of conservation observed between human and baboon miRNA sequences using *in silico *analyses, we used a human miRNA microarray to identify miRNAs expressed in baboon liver and lymphocytes. We then validated the expression of a select number of miRNAs using RT-PCR. Moreover, we determined the expression of the selected miRNAs in tissues relevant to dyslipidemia and cardiovascular disease.

In this study we identified and quantified miRNA using a combined approach of computational analysis and miRNA array. Of the predicted baboon miRNAs (N = 555), 40.9% sequences were identical to human pre-miRNAs. This is similar to 38.1% reported for rhesus macaque [37] and is consistent with alignment of DNA sequences between macaque and baboon showing on average 98% identity [38]. miRNAs (N = 494) were expressed in baboon liver and lymphocytes. The expressed miRNAs include miR-133, 208, and 21, which have validated targets for cardiovascular system [13]. We also detected expression of miRNA-335 and 122, which are associated with lipid metabolism [19]. Of the 494 expressed miRNAs, 58.5% were not predicted through *in-silico *analysis. This fraction may constitute new miRNAs not available in the current baboon draft genome assembly. Moreover, 350 miRNAs were predicted through alignment of human miRNA sequences with draft baboon genome sequences, but were not expressed in the miRNA array. Possibly this is due to tissue specificity of the miRNAs or miRNA expression below the detection limit. Completion of the baboon genome sequence, anticipated at 6× coverage, will enhance the prediction of putative miRNAs.

Previous studies have demonstrated that while miRNAs are conserved across many species, the expression pattern may be lineage and/or tissue/cell specific [39,40]. Of the total 494 expressed baboon miRNAs, 13.8% were expressed only in the lymphocytes, while 1.6% were detected only in the liver. We confirmed by RT-PCR that some miRNAs such as miR16-1 show more restrictive expression patterns. Further the RT-PCR results validate the results of the microarray assay; miRNAs that were expressed in the array were successfully amplified by RT-PCR and vis-à-vis miRNAs not detected. Moreover, expression of some miRNA is species-specific. While miR648 and 373 were reportedly expressed in the rhesus liver [37], these miRNAs were not detected in baboon liver using miRNA arrays or RT-PCR. Altogether we confirm previous evidence that miRNAs exhibit spatial and species-specific expression patterns.

A new class of unconserved miRNAs, existing in clusters, has been identified in many species. A miRNA cluster is defined as miRNAs exhibiting the same orientation and not separated by a transcriptional unit or a miRNA in the opposite direction [41]. Two large miRNA clusters on human chr 19 (N = 54) and X (N = 10) are conserved between human and chimpanzee and are specifically expressed in placenta and testis [42]. We sought to determine if these clusters are conserved in baboon as in other primates. In addition, we investigated whether the cluster members are expressed in baboon liver and lymphocytes. We observed that miRNA clusters on chr X and 19 are primate-specific and are conserved between human, chimpanzee, rhesus and baboon genomes. The absence of these clusters in non-primate species including rat and mouse indicates recent evolution in the primate lineage. Integration of the baboon draft genome http://genome-test.cse.ucsc.edu/ with previously published baboon linkage map [43,44] and comparison with the human and rhesus genomes shows complete synteny among human, rhesus and baboon for chromosomes X and 19. In contrast a smaller adjacent miRNA cluster (miR371, 2,3) on chr 19 (58,983000 - 58,983500 bp) is conserved in human, rhesus and rat, but not mouse [37]. In addition Yue and colleagues observed that miRNA clusters on chr 4 and 13 are also conserved in human, rhesus, rat and mouse. We observed conservation of these clusters in the baboon genome (data not shown). Altogether, this information suggests that while a subset of miRNA clusters are primate specific, some miRNA duplications occurred before the divergence of primate and rodent lineages.

Previous studies have reported that miRNA clusters exhibit linked expression patterns, suggesting shared cis-regulatory elements, and/or a polycistronic transcription [45]. This observation of linked expression patterns was affirmed in this study. For example, miRNA cluster members on chr 19 were down regulated while miR302a and 302d gene family members on human chr 4 and miR17, 18, and 19 on chr 13 [37] were expressed in baboon liver and lymphocytes. This observation suggests that expression of some miRNAs is closely linked via coordinated regulation of transcription rather than post-transcriptional modification or stability. Further, we noted that miR-514, a cluster member on the X chromosome was differentially detected in baboon liver and lymphocytes. Interestingly, miR-514 is known to have different copy numbers among primate species; three copies in human, four in chimpanzee and one in other primates [46], an indication that miRNA duplications may exhibit an evolutionary temporal pattern.

## Conclusion

We have used a combined approach of computational prediction and microarray analysis to identify and quantify baboon miRNAs. A search of homologous human pre-miRNA sequences in the draft baboon genome sequence (2× coverage) predicted 555 baboon miRNAs. miRNAs (N = 494) were expressed in baboon liver and lymphocytes using a human miRNA Beadchip. Of the 494 miRNAs expressed on the array, 41.5% were predicted by bioinformatics analysis indicating more than half of the expressed miRNAs were not predicted. This observation may be attributed to the status of the draft baboon genome sequence and that use of a microarray from a closely related species is important to discovering miRNA genes of an unannotated genome.

Our discovery of baboon miRNAs will provide resources for studies on the roles of miRNAs in dyslipidemia and atherosclerosis in tissues not accessible in humans. In addition the discovery of miRNAs in lymphocytes, which are easily accessible in humans, will be fundamental for translational studies.

## Competing interests

The authors declare that they have no competing interests.

## Authors' contributions

GMK, JPG and LAC participated in the conception and design of the experiments. LAC carried out the *in silico *prediction and analysis of miRNAs. GMK and JPG performed the experiments and data analyses. All authors contributed to writing this manuscript and all have read and approved the final manuscript.

## Supplementary Material

Additional file 1 **Primer sequences**. Information on primer sequences used to perform RT-PCR including the miRNA-specific forward and RT primer sets and universal reverse primers. Click here for file

Additional file 2 **Predicted baboon miRNAs**. Information on predicted baboon miRNAs including names, genomic coordinates, length, mismatches and percent identity with human pre-miRNA sequences. Click here for file

Additional file 3 **miRNAs array data**. The file provides information on Illumina symbol, target ID, normalized expression intensity, detection p-value, chromosome localization, probe and mature miRNA sequences. Detection p-value is calculated by the BeadStudio to determine significant level of expression intensity above baseline. For this study, p-value threshold was set at 0.05. miRNA with a p-value below the threshold was considered expressed. Click here for file
